# "Contagious Love": A Qualitative Study of the Couple Relationships of Ten AIDS Carriers

**DOI:** 10.2174/1874613600802010058

**Published:** 2008-08-08

**Authors:** Hadas Doron, Noa Teichner, Adi Grey, Yehudit Goldstein

**Affiliations:** Tel-Hai Academic College, Israel

**Keywords:** AIDS carriers, single-sexual couples, intimacy, passion, commitment.

## Abstract

The qualitative study in this article portrays the couple relationship among AIDS carriers, based on Sternberg's triangular love theory (involving domains of intimacy, passion and commitment). The central study hypothesis is that certain components of the Sternberg model will be more significant than others among the AIDS carrier population. The study was conducted on ten AIDS carriers aged 21-37 who had experienced a couple relationship. Six men and four women participated; most of them were in a romantic couple relationship of homosexual orientation.

The interviewees answered a questionnaire that included the three domains-- intimacy, passion and commitment--in the personal interview technique. The interview focused on interviewee's attitude towards his/her relationship with a partner, as he/she understood it. The findings of the study focus on relevant content that was gathered from the interviews and these portray a limited view of couple patterns in the world of AIDS carriers. The study reveals two major findings regarding the carrier's desires: On the one hand, the carrier describes a powerful need for a stable, permanent relationship--from the diagnosis of AIDS and throughout the subsequent years. On the other hand, the carrier also expresses powerful sexual desires that are not necessarily limited to a permanent partner. Thus passion is the dominant among the three domains.

The intimacy domain is mainly affected by disclosure of the disease and the joint coping that follows.

The findings are discussed in the context of the romantic internalized model theory and Sternberg's triangular love theory.

## INTRODUCTION

Much has been written about the effects of AIDS on various spheres of life. However, one aspect that has been relatively neglected is the impact of AIDS on couple relationships. AIDS is an inherently anti-couple disease as it is transmitted *via *sexual relations, thus couplehood is directly affected by the disease. We chose to enlarge the research field on this subject.

The literature review below relates to AIDS and its effect on couplehood.

## AIDS AND COUPLEHOOD

Much attention was focused in the past on the functioning of a family unit in which one member suffers from chronic illness or disability, but the functioning of the spouses as a couple has not merited sufficient attention.

The starting point for examining the effect of illness on a couple is the knowledge that the situation mandates a process of adjustment on the individual, couple and family levels. This adjustment takes place in the short-term as well as in the long-term--in an ongoing, broad sphere of coping mechanisms. The physical, psychological, social and economic impacts of the disease can make it difficult for a couple to maintain a stable relationship and keep the lines of 

communication open. The need for nursing services in the home can add even more severe, unique challenges. These sources of stress can have an adverse effect on the cohesion of the couple's relationship, on its stability and on the measure of satisfaction of the partners. Physical limitations can also disrupt the smooth running of the family and cause tension in a marriage. The effects of chronic illness can include ongoing deterioration in mobility, employment and earning a livelihood; escalating treatment costs; continuous weakening in the physical, emotional and social realms and difficulties in sexual functioning.

The partner or spouse of a person with a disability or disease must acquire crisis-managing and coping skills without any advance preparation and then practice them under cumulative conditions and over an extensive period of time. These kinds of situations lead to changes regarding role division of partners, quantity of tasks, expectations of functioning, and emotional participation. Sometimes these changes are accompanied by a buildup of care-giving tasks that may tax the healthy partner to the maximum vis-à-vis his or her physical, emotional or economic abilities. Furthermore, these changes create gaps between the partners regarding their evaluation of the condition, their expectations of coping with the situation and the assessment of the other spouse's method of coping. These changes take place in both the inter- and intra-personal realms [1].

The challenges described above are even more salient when one of the spouses suffers from AIDS, a contagious disease transmitted by sexual relations which has great impact on the relationship of the couple. Yet despite the greater public awareness of AIDS and dissemination of the ways to prevent its transmission, there has not been a decrease in the number of newly infected AIDS victims every year. In fact, many countries report a yearly rise in the number of new incidents. One of the possible explanations for this phenomenon may be attributed to the unwillingness of AIDS carriers to use condoms as they perceive this as a form of social coercion that impinges on their enjoyment of sexual relations [2, 3]. Another reason seems to be the viewpoint among homosexuals in which the contraction of AIDS is a "calculated risk," reflecting typical sexual behavior among homosexuals [4].

Another theory attributes the world-wide phenomenon of risk-taking in sexual practices to misguided optimism regarding the prognosis of AIDS, an optimism that leads to complacency regarding taking appropriate measures of 'safe sex' [4, 5]. Yet another study that was conducted among homosexual couples in which one member was an AIDS carrier, revealed that between a quarter to one-half of the participants did not practice preventative measures ('safe sex') on a regular basis [6]. A study in the United States found a correlation between depression caused by AIDS and high-risk behavior among women with AIDS [7], And according to Armistead *et al* [8], women affected by AIDS-related depression tend not to disclose their illness to their sexual partners.

Couplehood in general contains the seeds of conflicts. When AIDS becomes another variable in a relationship, it tends to be an additional source of conflict and often leads to violence among the spousal members [9]. Spousal conflict has a significantly negative effect on the emotional health of women with AIDS since the disease itself affects the physical, economic and social needs of the women [10]. These special needs may heighten the vulnerability and dependence of the women within their domestic relationship. In addition, AIDS can distort the natural quality of the couple relationship. A study conducted by Milan and his colleagues [11] found that AIDS can change the sexual patterns between spouses while affecting the level of intimacy. The study examined couples in which both spouses were AIDS carriers and found that while some couples reacted in anger by flinging mutual recriminations at one another, other couples treated their joint fate as a source of significant mutual support. Still, it was found that many women with the disease tended to remain within conflict-ridden relationships as they felt that their disease limited their chances of finding better partners [11].

A study in the United States found that homosexuals not in a committed relationship who practice unprotected sex tend to see life pessimistically, express dissatisfaction with their lives and predict a shorter life-span for themselves. On the other hand, AIDS carrier men who practice safe sex and men who are in stable relationships are more optimistic about their disease and express greater satisfaction with life. The research found that these findings are congruent with the parallel situation among the heterosexual population [12].

Not enough research has been conducted (both in Israel and throughout the world) regarding the coping of spouses living with the AIDS virus. While couple relationship in general is viewed as an important developmental stage in the human life, AIDS is by essence an anti-couple disease due to the fear of contagion from sexual relations. The current study examines how these two factors interact.

This study examines three domains of couplehood: passion, commitment and intimacy--as they are expressed by AIDS carriers within the existing (or desirable) spousal relationship, according to Sternberg's triangular love theory [13], which views love as a triangle whose vertexes are intimacy, passion and commitment. In contrast to other characteristics of love, which are limited to a time period or specific culture, these characteristics cross the boundaries of time and place.

**Intimacy**--refers to emotions that arouse closeness, warmth and mutual interrelation among spousal members. The major attributes necessary for intimate love include trust, honesty, respect, commitment, and security.

**Passion**--this component is connected to a strong longing for full union with the loved person. Passion is an expression of needs and desires such as the desire for self-esteem, nurturing and belonging. When we think of 'passion' we tend to think, first and foremost, of sexual passion but any form of psycho-physiological arousal can create passion.

**Commitment**--There are two aspects of the commitment element in love. The short-term element is the decision to love someone, and the long-term one is the commitment level of the relationship. Usually the decision to love precedes the decision to commit.

The importance of each of the components of love differs from short- to long-term relationships. While passion is a central component of the short-term relationship, intimacy and commitment are more important to the long-term union. In addition, the degree to which each of these components is present, changes from relationship to relationship. Intimacy is salient in many loving relationships, passion characterizes romantic unions, and commitment tends to vary greatly from relationship to relationship.

While all "love triangles" have three mutual, permanent above-mentioned vertexes, each of these vertexes can sprout additional 'triangles.' Thus a relationship can encompass a number of different love triangles which differ in their magnitude (degree or extent of love), their configuration (balance of the love components), whether they represent the existing relationship of the couple (realistic relationship) or the aspirations of the spousal members and what they hope to achieve (the ideal relationship)-- and finally, whether the triangles represent emotions or actions.

Finally, while an equilateral triangle represents a balanced love in which all three components of love are roughly equally matched, a scalene triangle pointing to one side, represents either a relationship in which the passion component of love is emphasized over the others – this relationship is likely to be one in which physical attraction plays a large part; or a relationship in which the decision/commitment component predominates over intimacy and passion – it is the highly committed relationship in which intimacy and physical attraction have waned or in which they were never there in the first place. An isosceles triangle, on the third side, represents a relationship in which the intimacy component plays a large part and the passion and decision/ commitment components play smaller parts. This triangle represents a relationship in which the two lovers are very good friends and are close to each other but the physical aspects and commitment to the future are more marginal [14].

It seems that no study had examined the effects of an 'anti-couplehood' disease such as AIDS on existing and desirable couple patterns. Thus we view this study as a significant contribution to the body of knowledge in the field of love relationships.

## RESEARCH METHOD

The qualitative study facilitates in-depth understanding of procedures/behavior and phenomenological meanings out of a human worldview composed of a multiplicity of subjective realities [15, 16]. The purpose of the researcher is not to corroborate or refute theories but to study, analyze and conceptualize the different interpretations of multi-realities [17, 18, 19]. In the context of the present study, the general question is: "What is the overall picture of existing couplehood among AIDS carriers in Israel according to Sternberg's three dimensions of love: intimacy, passion and commitment?"

The questions comprising the interview used in this study are based on the "The triangular love index of Sternberg" [14]. Sternberg used this questionnaire to measure the amount of love in one of the studies he formulated to support his triangulation theory.

### Participants

The population study included 10 interviewees, all AIDS carriers. There were six homosexual men (aged 21-46) and four heterosexual women (aged 23-25). At the time of the interview, 5 interviewees were in a spousal relationship while two of them were each other's partners. The interviewees were aware of their status as AIDS carriers for a period of time varying from 7 months to 17.5 years.

### Data Collection Process

The researchers disseminated questionnaires at a yearly AIDS convention *via *the Israel AIDS Task Force****(IATF). Although there was initial willingness to fill out the questionnaires, the examinees quickly lost their enthusiasm and said that the "questionnaire is not relevant to us, it is too long and tiring [to fill out] and is not appropriate to the homosexual population." As a result, only three full questionnaires were returned and the unexpected need arose to change the structure and wording of the questionnaires and to recruit a target population. Thus it was decided to adopt the qualitative research method. From that point on, recruitment of examinees was carried out *via *the "snowball method."

The interviews were carried out in the offices of the IATF (except for one interview held in the house of an interviewee). The interviews were about two hours in length each and were recorded and transcribed by the interviewers. Participants who were not involved in a relationship during the time of the interview were asked to talk about a former relationship. The head researchers conceptualized the following five main headings for the interview: the behavior and feelings that accompanied the disclosure/diagnosis of the disease; the disease as a secret; intimacy, passion and commitment.

### Data Analysis

The researchers analyzed the interviews separately to uncover major themes, using constant comparative method, then held a discussion to synthesize the themes while using grounded theory principles [20].

Content analysis was carried out in four major stages:

### First stage:

Reading all the interviews to become closely familiar with the words, outlooks, thoughts and emotions of the interviewees [21].

### Second stage:

Identifying, dividing, sorting and organizing of repeating sections connected to study issues, and/or those sections that have broad qualitative influence and ramifications for the interviewees [22]. Parallelly, themes begin to be developed.

### Third stage:

Creating core categories by reducing the number of categories to the following five:

A. Behaviors and feelings when the disease first made its appearance.

B. The disease as a secret: variables of the disease as a secret.

C. Intimacy: Emotional communication and the degree of trust in the relationship.

D. Passion: Attraction, sexual behavior and fantasies regarding the partner.

E. Commitment: stability of the relationship and sexual commitment.

The findings are presented below according to these categories and are illustrated by representative quotes from interviews (a fictional name of the interviewee appears at the beginning of each quote).

## RESULTS

An attempt was made to comprehensively cite the participants' perspectives regarding the impact of the AIDS virus on the three variables: intimacy, passion, commitment..

The research findings are distilled into five major themes that emerged from analysis of the data.

### Behaviors and Feelings that Arose when the Disease was First Diagnosed

The diagnosis of the AIDS virus brings on painful/difficult feelings of loss, terminality, severe emotional shakeup and attempts at repression. All the interviewees viewed the disease as something that would change their lives in a negative, extreme manner:

Ami: "In January I received the results and my life was turned upside down, I had very difficult moments…what we knew then was that people [with AIDS] died from the disease. My 35th birthday was like a funeral, family and friends 

came to say their goodbyes."

Talya: "At the beginning of the relationship I felt 100% perfect, but ever since the disease I don't feel that way…Sometimes I cry, why did it have to happen to us, why me? Sometimes I cry and think that I'll never be free [of this ill ness] my entire life."

Yet after the initial emotional storm that accompanies the disclosure of the virus, we see that most of the interviewees then describe feelings of desire to re-build their lives, to continue to function and even actualize themselves:

Eran: "Three days after my diagnosis I was released from the army and started to work as a waiter…I viewed it as a personal achievement that [I developed aspirations] for the remainder of my short life: I decided that I wanted to be part of a long, stable, productive relationship; after that, I could die [in peace]".

### The Disease as a Secret: Variables of the Disease as Secret

AIDS victims are often the objects of widespread, negative social stigma; therefore many of the interviewees were initially reluctant to disclose it to partners or family members.

Talya: "I didn't tell anyone, they think I have low iron and that's why I'm weak. Even my older sister doesn't know…I don't even buy the medications myself, I can't bring myself to get near the place. I send my boyfriend and

he makes the purchases. I don't want to see that place and don't want them to see me".

Yet when the person made the decision to confide in certain people about the AIDS diagnosis, all the interviewees received support and recognition:

Oren: "I was very afraid of what would happen if they'd know, but all my friends know and no one turned their backs on me. Anyone who won't accept me, won't accept anyone."

The IATF and the community of AIDS carriers are sources of significant support in the lives of the interviewees. The support is expressed in the transmission of knowledge, in physical and emotional backing, and help in the couplehood sphere:

Shiri: "The first thing he did was take me to the IATF. At first I put my foot down, but he dragged me there. I sat here with a few representatives and talked to some people and also with the social worker. I emerged like a reborn person. Suddenly, things opened up for me, I felt different."

An important issue raised by most of the interviewees is connected to the process of revealing the AIDS secret in the couple relationship. The main difficulty stemmed from fear of rejection and the dilemma was: at what stage to tell the partner about the AIDS virus--if at all?

Eran: "Some people don't tell anyone. For a year they take their meds in private. One of the central questions is, how and when to tell? Before sex? After sex? How to bring it up after a month? Coping with repression. It's a matter of trial and error, to size up your partner as quickly as possible, how he'll cope with it. Will he run away, reject you, become violent, betray your secret to others, etc."

## INTIMACY

### Emotional Communication

Concern for the well-being of the partner was expressed in all the interviews. Evidently AIDS is a significant variable in relation to emotional communication, due to the health-related aspects of the disease:

Eran: "I worry a lot about his well-being. On his side, there's room for improvement. He does take care of my medications, he brings me the pills when I ask. As far as coping with actual illness, till now we haven't had to deal with that. He's happy when I get good results [from the blood tests] and we even drink a toast to that. So [he] is concerned about my welfare, but in a different way.

Oren: "I'll always feel great responsibility for him. If he doesn't work then I take care of him, when he's sick I make sure he takes his meds, or things connected to his family."

Most of the interviewees mentioned that the mutual coping of a couple (when one of them has AIDS) bestows a measure of security on the relationship and alleviates the daily difficulties associated with the disease:

Eran: "Joint coping makes it easier to deal with the meds. There was an experimental treatment involving eggs that was smelly and disgusting. Since we were together, I coped a lot better than if I had been alone. He used to prepare the medicine for me and we would take it together. It was a very unpleasant treatment and being a couple helped."

Oren: "Ro'i helped me know more [about the disease]; we'd go to the doctor together, I'd nod my head and afterwards Ro'i would explain to me [what the doctor said]. That's his commitment--he makes sure I take my pills and meds."

In addition to solicitude, welfare and emotional support, 5 out of 6 interviewees testify to difficulties in communication and a perception of lack of understanding on the side of the partner:

Oren: "Ukrainians are emotionally blocked. You're not supposed to talk [with them] about problems or difficult things. Every time I try to raise issues about things that are difficult for me, he treats me like a weakling, someone who goes to emotional extremes. It's hard to tell what he thinks or feels. They know nothing about talking about problems in order to understand the person they live with. There is an emotional block at the foundation of the relationship. I don't know everything about him."

Oren: "During the recent time period I feel that I'm not receiving enough emotional support. I don't [the reason], but something is missing. Our communication could be better. Often we don't understand each other, for example when he answers me in a certain tone of voice it creates misunderstanding, also the reverse is true. Our communication is not working well, we came from different family backgrounds--in my house it was acceptable to shout, but not in Ro'i's family.

### Degree of Trust in the Relationship

The degree of trust is described by the interviewees as comprised of variables such as: security and honesty in the relationship, sharing experiences and problems, friendship and esteem. Most of the interviewees interpret 'trust' on two

Eran: "He gives me security that he is with me and will remain with me. He stays with me because he wants to be with me; not because of convenience or because it's a great honor."

Oren: "I can rely on him that he will remain in the relationship even in difficult periods, let's say if I'll be in the hospital then I know he'll be there for me."

Some interviewees said that the disease interfered and inhibited the formation of trust in the relationship. Shiri describes her desire to keep the illness to herself versus the need to involve her partner fully in her life as two contradictory needs that create conflict on the subject of trust:

"I put up a wall in front of him [It came to the point where] either I'd talk about what's happening, or it's over. I felt that our relationship was starting to deteriorate. One day, after a year, I got up the courage and told him. I didn't know how he would react…It was to terribly stressful…But the first thing he said was, 'Darling, I'm so sorry.' See, he thought about me first! Then I cried a little and the penny started to fall in his mind: 'Why didn't you tell me? How could you assume responsibility for me? You know that we are not in a committed relationship, how could you take responsibility for another girl that maybe I was with."

## PASSION

### Attraction and Sexual Behavior

An important and essential variable that figured prominently in the interviews is mental and physical preoccupation with sex. All the interviewees described great sexual attraction and passion in the couple relationship:

Oren: "I very much enjoy physical contact. There's something magical when he touches me, the palm of his hand [touches himself], one like that and you're in the clouds…"

Ro'i: "I'm always horny over him. We always want to be together but that feeling can disappear for a day or two when we're irritable at each other. I get excited when I see him, I think about him during most of the day. I fantasize about him a lot."

Ami: "There's a lot of passion between us. I get very excited over him. Sometimes I feel like a teenager. Our ability to encourage one another surprises me. Like two children."

In most of the cases, this passion is also expressed in sexual openness, unique sexual experiences and externalized sexual behavior.

Eran: "He compliments me that I'm the best lover he ever had, and we enjoy sexual experiences beyond just the two of us (with a third person). I enjoy participating in novel [sexual] experiences for him."

Ami: "Sometimes we get carried away. Yesterday when we came home we found ourselves running wild in bed; usually we close the door but this time we didn't notice that our daughter had come home…it was very funny…we didn't stop laughing."

Still, half of the interviewees talk about sexual problems arising from the existence of the HIV virus. Note two major points in this context: the danger of infection of the virus via sexual relations, and the danger posed to the HIV carrier from the introduction of additional germs in the body (resulting from damage to the carrier's immune system):

Eran: "There's lack of sexual satisfaction with people (in my present relationship there is great sexual satisfaction, I'm talking about other relationships). Couples in a relationship expect to be able to stop using a condom at a certain point, and entering a relationship with a carrier carries with it the commitment to using a condom 100% of the time."

Talya: "Regarding sex, at the beginning everything was great, we couldn't do without it. We'd do it several times a day. Ever since the disease, it's not all the time. He says that he doesn't feel like a man completely because he can't enjoy it."

Ami: "One more infection isn't enough to affect our lives, we can be together completely freely. That's the choice of two adult men. I can understand people who are not willing to make such a decision and decide they don't want to be infected by the virus and so they use condoms all their lives. Everything is possible. People make their own, personal choices. I don't think there's a consensus on this issue."

### Fantasies About the Partner

All the interviewees said that they fantasized about their partners. They also talked about feelings of great admiration and appreciation:

Ro'i: "I feel that our relationship is enchanted, magical. I admire him for many things--stubbornness (like me), for things he does despite the difficulties involved… personal responsibility. I am capable of imagining life without Oren but such a life would be boring and routine."

Ami: "Sometimes I admire him. He has tons of strength and abilities in things that I can't do. More than that, though--I appreciate him… I admire his ability to contain things in life, but I am also very critical about a lot of things. There are things I wish would change about him but I knew that there are things that won't change, just like it's hard for me to change."

Talya: "I think he's perfect."

## COMMITMENT

### Stability of the Relationship

The stability of the couple relationship encompasses two intertwined aspects. Half of the interviewees described the stability of their relationships on the day-to-day, here- and-now level, and how it affects commitment in the future:

Ro'i: "I am thankful for his existence and feel that I can truly trust him. I know that if I run into a problem I have someone I can lean on, for example in the recent period regarding financial matters, or when I was hospitalized and he was with me."

When facing the future, 4 out of 6 interviewees felt difficulty in being secure about the stability of the relationship:

Ami: "I felt that since I don't have much time to live while my partner has his whole life ahead of him, I should break up with him. Afterwards I realized that's a silly way to think-- neither of us really knows how much longer he will live."

Eran: "Commitment both stabilizes our relationship and also undermines it at the same time. It's hard to explain. On the one hand we try to be stable and cope with very difficult situations, but sometimes we feel that we're on a roller coaster. We have a lot of ups and downs."

Ro'i: "I really care about Oren, I have confidence in the stability of our relationship but I don't want to think what will be, I just let things happen. I hope that things will remain as they are now and that my love for him will remain…I view my commitment for mutual understanding and a stable, unshakable relationship, that is what will ensure the stability of our relationship. I can imagine a scenario in which the relationship will end, but I am sure of my love for him. I don't think ahead 10 or 15 years, that's too far, but I do see our relationship as a permanent thing."

Most of the interviewees express the desire and willingness to struggle and sacrifice for the stability of the couple relationship. The sacrifice is expressed by forfeiting contact with the family of origin in favor of the partner, by relinquishing a livelihood, giving up old habits and even risking damage to one's self-respect:

Eran: "I'll fight to maintain the relationship, I am willing to swallow my pride, I'll do everything I can. There was a time when he wanted to leave, I begged him and even went down on my knees. I'll do anything, I believe that's the right thing--up until murder (laughs)."

Oren: "In principle I'm willing to give of myself. That means to give up certain things like family, to risk things in other areas for the relationship and for Ro'i…Work, for example? So I'll find other work. Work is important… but it's more important to me that it should be good for me with Ro'i."

Ro'i: "I feel that the relationship with him is the most important thing in my life, it expresses sacrifices I am willing to make. Sacrifices for the man I care about, even crucial things like not going alone to parties… this is an example of a sacrifice I make for him."

One interviewee describes his commitment for his partner in the fact that he encourages his partner to assume responsibility to persist in taking his medication, in order to lengthen the time he will have with his partner in the relationship:

Eran: "This love is so great that it causes me to assume greater responsibility for my health and my meds in order to prolong this love. Before I met Sasha I had big problems in following my treatment plan, I put down the whole pill scene and played games with them. Now I'm a lot more careful; it's part of commitment, he also says so--to remain healthy for as long as possible."

## SEXUAL COMMITMENT

Most of the interviewees do not feel sexual commitment (fidelity) towards their partners. This lack of commitment is expressed in two ways: by sexual experiences with other partners, as well as by introducing other partners to the sexual act between the couple:

Oren: "I am not committed to him when it comes to sex. As a rule it doesn’t bother Ro'i because he knows that I distinguish between sex and true love, and it also doesn’t bother me, but I'm not willing that Ro'i won't be committed to the relationship."

Ami: "We have an open relationship, which means that if one of us meets someone else, it's OK to go with him. We can also be three, or with another couple. It's not a way of life but if it happens, we don't make a big deal about it. It doesn't threaten our relationship. That's the understanding we have and we really want to be together. It's not instead of, but in addition to."

Eran: "We like enjoying sexual experiences beyond the two of us, with a third person."

One interviewee described the fact of her being an AIDS carrier as the factor that harmed her willingness and emotional ability to assume a committed, revealing couple relationship:

Shiri: "Here and there it continued as an uncommitted relationship. It was convenient for him too, he was afraid of commitment, but that was his business and his fears. As far as I was concerned, it was very convenient. I didn't have to tell him what was happening with my body. It was convenient for me to tell him that I don't take [birth control] pills and I prefer a condom."

## DISCUSSION

This study deals with significant issues regarding the relationships of AIDS carrier couples in Israel. The findings point to a picture of couple relationships involving elements affected by AIDS as well as elements reflecting normative couples, in accordance with Sternberg's love indexes: intimacy, passion and commitment.

Below are some conclusions drawn from an analysis of the findings based on Sternberg's triangular theory of love:

### Intimacy

The study hypothesis was that the variable of 'intimacy' would be more dominant among couple relationships with AIDS sufferers or carriers whose medical situation was known, in comparison with couples in which the disease was kept secret. This hypothesis was strengthened. According to Clark &amp; Reiss [23], intimacy is defined as a process wherein a person expresses emotions and conveys important, relevant information about himself to another human being. This process facilitates the building and strengthening of one's personality and is a part of one's basic human needs. According to Segel-Englizhin [24] and most researchers, personal exposure--involving revelation of one's feelings and important personal information to another--is a necessary and sometimes even necessary-and-sufficient condition for intimacy to take place. Many theories emphasize the decisive influence of intimacy on the individual, especially on one's mental health [25]. Furthermore, intimacy is an important component of happiness, of imbuing meaning in life, and of deriving satisfaction in a relationship [26]. This study's findings corroborate these definitions. The interviews showed that mutual, honest coping with the AIDS illness provides security and alleviates the day-to-day difficulties that accompany the disease. When the illness is kept under wraps, as one of the interviewees describes, the relationship cannot develop and deepen. Instead, the 'secret' becomes a barrier for the development of intimacy in the relationship.

## PASSION

It seems that the research hypothesis whereby passion would have less importance among AIDS carriers, has been refuted by the study. On the contrary: the passion variable emerges as important and fundamental amongst all the interviewees who described strong sexual attraction and passion in their couple relationship. In most cases this passion is expressed by a readiness for unique sexual experiences and externalized sexual behavior. A possible explanation for this interesting finding may lie in the fact that most of the interviewees belong to the homosexual community which has different sexual patterns than the heterosexual world, as mentioned at the beginning of the chapter.

Foucault [27] claims that sex has become "the truth at the foundation of our existence" that is--the central axis in our self identity. This viewpoint is reflected in the homosexual culture where sexuality is salient and is testimony to quality of life. Among some male homosexuals, sexuality is important in self-identity and self-esteem. When the individual copes with a psychological threat, he needs some kind of defense to stabilize the emotional jolts and, thus, uses sexuality as strategic compensation, playing a central role in the coping process. The importance attributed to sexual relations can serve as a distracting mechanism from stress, anxiety and depression and raise one's self image [28].

Another explanation for the centrality of sex in the lives of the interviewees is the initial development stage of their relationships. According to Sternberg [13], there are different types of love that characterize different stages or periods of the couple's relationship over the years. At the beginning of the relationship there is usually a correct, balanced integration of the three components of love. Most of the interviewees in the study are at the stage of building their relationship, which is different than being partners in long-term unions. In the love that characterizes long-term stable unions, physical attraction tends to fade thus passion takes a smaller role.

## COMMITMENT

The research hypothesis regarding commitment among AIDS carrier couple relationships was that the commitment component would receive less importance than the other two elements in the model.

The research presents two central findings related to security, stability of the relationship and trust on the one hand, and sexual connection on the other: While the interviewees describe a strong need for a stable, permanent relationship--from the diagnosis of AIDS and throughout the years--the need for sexual contact is not necessarily limited to the permanent partner but is very powerful and externalized.

A possible explanation of this duality may be found in psychoanalytic object-relation theories. The concept behind these theories is that the mother-infant relationship is internalized by the child and becomes a model to the child for other relationships during his life [29]. Hendricks [30] claims that the choosing of a spousal partner is done according to deep psychological needs that are internal and subconscious. According to this approach, the individual selects a spouse according to the traits of his/her parents in order to re-experience the relationship, as an adult. In falling in love and in choosing a partner we reconstruct a relationship with intense emotions including closeness, longing and dependence yet unavoidably and unconsciously, this relationship will also include old frustrations and repressed feelings [31]. It is possible that the need for a stable relationship, as expressed in the interviews, is in effect an attempt to revive the care-taking figure--the mother--especially in light of AIDS unique medical needs and the object-relation internalization of the AIDS carrier.

The findings demonstrate that AIDS carriers often develop the desire to create deep, stable and significant relationships for the rest of their lives. While all couple relationships have a component of searching for a parent figure, the need for medical attention amongst this specific population increases and intensifies the search for a stable, caretaking figure. In effect, there is a hidden conflict between knowing that one's life expectancy is likely to be shorter than usual, and the need to devote oneself to a couple relationship. A shorter life expectancy leads the carrier to the desire to make the most of life. Most of the interviewees reported that they do not feel sexually committed to their partners. Perhaps the AIDS carriers' desires to 'make the most of life' is expressed by their experiences of multiple sexual partners and experiences.

The findings demonstrate a clear distinction in sexual commitment between homosexual and heterosexual interviewees. Therefore, we must avoid assigning false labels on sexual behavior as being normative among AIDS couples. It is likely that this behavior is part of accepted sexual patterns in the male homosexual community as a whole, and not necessarily the homosexual AIDS couples in specific.

There are some who claim that social isolation of AIDS carriers leads to lack of support of the victims in their communities [32]. The study findings weaken this claim and shows that support networks among the population of homosexual AIDS carriers is significant and encompasses many spheres of life. It seems that there is great openness and acceptance in this community towards carriers, allowing them to become integrated and receive meaningful social support in comparison to the social isolation reported by the heterosexual (female) interviewee. Furthermore, there seems to be a connection between the social support finding and readiness to disclose the illness to the surroundings. Coming public with the disease is a complicated, conflictual process that received much attention from all the interviewees. The central dilemmas revolve around fear of rejection and ostracism. The homosexual interviewees displayed much greater openness and willingness regarding exposure. Moreover, the heterosexual interviewees were suspicious and refused to reveal details that could give them away in the future and acted uncomfortable throughout the interview, as opposed to homosexual men. However, perhaps some of the differences can be attributed to the male-female divide, and not only the hetero- and homosexual one.

There are several reasons for the world-wide negative stigma attached to AIDS. First, the illness is identified with sexual relations, homosexuality, drug use and death. Secondly, incorrect notions regarding the transmission of the illness are still rife in the general population. As a result, many women with AIDS report outright social isolation [33], which in turn is related to a limited use of AIDS-related social services, in comparison with men who tend to use these services much more.

Additional significant differences are embodied in the health status of partners of AIDS carriers. The findings point to asymmetrical power division in relationships where one of the partners is not an HIV carrier. This situation seems to arouse feelings of inferiority among carriers and intense fears of abandonment. The carriers see themselves as undesirable, defiled and with low chances for creating a new couple relationship. Other studies conducted in the United States strengthen the results of this study and claim that the very existence of AIDS, causes many afflicted women to remain within conflictual relationships due to their belief that their illness limits their abilities for creating new spousal relationships. Furthermore, conflicts in the couple relationship can have significant negative impact on the mental health of women with AIDS since the illness itself affects physical, economic and social needs of women [10]. This fact may serve to explain the sense of dependence described by those interviewees in relationships with healthy partners.

## STUDY LIMITATIONS

It is important to address the major limitation of this study that affects the results and conclusions drawn from it: the study integrates both homo- and heterosexual interviewees, thus making it impossible to isolate the AIDS variable. The homo- and heterosexual couple populations have different lifestyles in certain domains, thus it is hard to attribute research findings to the presence of the HIV virus in a single-sex couple when the results are typical of the behavioral norms in the homosexual community; for example, multiple sex partners. Furthermore, the homosexual experience carries with it a unique gamut of psychological issues, and coping with HIV only adds to these already present issues. We feel that homosexual partners living with AIDS should be studied separately in their own unique sociological context, which is replete with negative stigmas.

Another limitation of the research is the small number of participants who agreed to take part in it. One possible cause is that the questionnaire was not appropriate to the target population (as described above). However, the lack of participation may also be partly attributed to the unwillingness or uncapability of desired participants to expose their couple relationship to foreign researchers.

## CONTRIBUTION OF THE STUDY

The study hypotheses and their results have great importance as they portray preliminary outlines of AIDS couple relationships--a population that has so far received very little attention both in research and in theory. This situation created great difficulty in finding theoretical material for the body of the work and as a basis for findings. We can even say that this study is pioneering in its sphere, and contributes to a body of knowledge in the subject of AIDS disease's influence over couplehood.

The research results may contribute to couple therapy of AIDS couples, by making us aware of the typical emotional reactions and stresses facing these couples. This study and additional ones in the sphere will form a basis for establishing both treatment techniques and preventive therapeutic programs towards creating open, aware, healthy and productive couple relationships.

This study opens a number of possible directions for future research. First of all, it is important to conduct comparative research between couples coping with AIDS and healthy couples in different couple configurations (homo- or heterosexual unions). This comparison could bring to light the unique coping domains of AIDS couples and contribute to the development of intervention and assistance methods. An important emphasis should be placed on isolating the sexual preference variable that crudely affects the results of the study. Second, the components of the commitment variable need to be studied more in-depth as it seems to be influenced by many independent factors (such as sexual orientation) and it would be interesting to examine which additional factors influence the evaluation of this variable in the couple relationship. Third, a longitudinal study could be conducted to examine the three variables of love according to Sternberg-- intimacy, passion and commitment--within couple relationships of AIDS carriers and then at a later stage, to examine these same domains in more advanced stages of full-blown AIDS. This kind of study would facilitate credible comparison of these domains at different points in the development of the couple relationship, and also enable close examination of the effects of the disease during active, decisive coping. In light of the findings of the present research, such a study would be extremely important.

## SUMMARY

The present study examined the relatively chaste field of AIDS carriers' couple relationship, based on Sternberg's triangular love typology [14]. After conducting qualitative interviews, It was found that AIDS carriers put much emphasize over both intimacy and passion, while displaying a paradoxical attitude towards commitment. However, these results should be considered pilot/explorative findings, paving the way for further and more comprehensive investigation.

In summary, we feel that there is great ignorance and lack of awareness regarding this disease and its results in the sphere of the couple relationship, making it evident that the subject of the couplehood of the individual coping with AIDS requires additional empirical research.
